# Twenty years of capacity building across the cancer prevention and control research network

**DOI:** 10.1007/s10552-023-01690-2

**Published:** 2023-04-17

**Authors:** Mary Wangen, Cam Escoffery, Maria E. Fernandez, Daniela B. Friedman, Peggy Hannon, Linda K. Ko, Annette E. Maxwell, Courtney Petagna, Betsy Risendal, Catherine Rohweder, Jennifer Leeman

**Affiliations:** 1 Center for Health Promotion and Disease Prevention, The University of North Carolina at Chapel Hill, Chapel Hill, NC USA; 2 Rollins School of Public Health, Department of Behavioral Sciences and Health Education, Emory University, Atlanta, GA USA; 3 School of Public Health, The University of Texas Health Science Center at Houston, Health Promotion and Behavioral Sciences, Houston, TX USA; 4 Department of Health Promotion, Education, and Behavior, University of South Carolina, Arnold School of Public Health, Columbia, SC USA; 5 School of Public Health, Health Promotion Research Center, The University of Washington, Seattle, WA USA; 6 Los Angeles, Fielding School of Public Health and Jonsson, Comprehensive Cancer Center, Health Policy and Management, The University of California, Los Angeles, CA USA; 7 Colorado School of Public Health, Department of Community & Behavioral Health, The University of Colorado Denver Anschutz Medical Campus, Aurora, CO USA; 8 School of Nursing, The University of North Carolina at Chapel Hill, Chapel Hill, NC USA

**Keywords:** Capacity building, Mini grants, Training, Implementation practice, Evaluation, Evidence academies, Online tools

## Abstract

**Purpose:**

To improve population health, community members need capacity (i.e., knowledge, skills, and tools) to select and implement evidence-based interventions (EBIs) to fit the needs of their local settings. Since 2002, the Centers for Disease Control and Prevention has funded the national Cancer Prevention and Control Research Network (CPCRN) to accelerate the implementation of cancer prevention and control EBIs in communities. The CPCRN has developed multiple strategies to build community members’ capacity to implement EBIs. This paper describes the history of CPCRN’s experience developing and lessons learned through the use of five capacity-building strategies: (1) mini-grant programs, (2) training, (3) online tools, (4) evidence academies, and (5) evaluation support for partners’ capacity-building initiatives.

**Methods:**

We conducted a narrative review of peer-reviewed publications and grey literature reports on CPCRN capacity-building activities. Guided by the Interactive Systems Framework, we developed histories, case studies, and lessons learned for each strategy. Lessons were organized into themes.

**Results:**

Three themes emerged: the importance of (1) community-engagement prior to and during implementation of capacity-building strategies, (2) establishing and sustaining partnerships, and (3) co-learning at the levels of centers, networks, and beyond.

**Conclusion:**

CPCRN activities have increased the ability of community organizations to compete for external funds to support implementation, increased the use of evidence in real-world settings, and promoted the broad-scale implementation of cancer control interventions across more than eight states. Lessons from this narrative review highlight the value of long-term thematic networks and provide useful guidance to other research networks and future capacity-building efforts.

## Introduction

The Cancer Prevention and Control Research Network (CPCRN) is a national thematic network of academic, public health, clinical, and community partners who study the implementation of evidence-based interventions (EBIs) to prevent and control cancer [1]. With funding from the Centers for Disease Control and Prevention (CDC), CPCRN supports implementation of cancer control EBIs in clinical and community settings, with a focus on settings that reach those at greatest risk for health disparities (e.g., clinics, health departments, and community-based organizations) [1]. The CPCRN network includes Prevention Research Centers [2] at universities across the country, each of which has their own regional networks of community, academic and healthcare partners; conducts its own research projects; and collaborates in cross-center workgroups. Throughout most of its 20-year history, CPCRN center projects and workgroups have included a focus on building community and healthcare partners’ capacity to implement EBIs, with capacity defined as the awareness, knowledge, skills, self-efficacy, motivation, and resources to adopt and implement EBIs [3]. In formative work, CPCRN members found that even when partners recognized the value of EBIs, few accessed EBI resources on the CDC or National Cancer Institute (NCI) websites [4]. When partners did implement EBIs, they were less likely to implement those that targeted system-level change and therefore limiting the potential to have substantial impact [5–7]. To address these gaps in capacity, CPCRN members reviewed the literature to identify effective capacity-building strategies [3] and began to develop theory to guide capacity-building initiatives [8].

CPCRN’s capacity-building work is guided by the Interactive Systems Framework [9], which posits that three levels of systems interact to promote and support EBI implementation. Synthesis and Translation systems summarize information about EBIs and disseminate them widely (e.g., NIH provides EBIs on its *Evidence-Based Cancer Control Programs* (EBCCP*)* website) [10]. Delivery systems adopt and implement those EBIs in practice (e.g., community clinics adopt EBIs from EBCCP) [10]. Because delivery systems often lack capacity to implement new EBIs, a third level of systems—support systems—is needed to provide training and other support to build delivery system capacity. Although the CPCRN performs the functions of all three systems [11], in this paper we focus on the CPCRN’s role as a support system that works to build delivery system partners’ capacity to select and implement EBIs. In this paper, we describe five strategies CPCRN has developed to close the gap in public health, clinical, and community partners’ capacity to implement cancer control EBIs. These strategies include (1) mini-grant programs, (2) training, (3) online tools, (4) evidence academies, and (5) support for evaluations of capacity-building efforts. We provide a brief history of the development and use of each strategy within and beyond CPCRN. We also provide illustrative case studies with lessons learned from across the network.

## Methods

**Fig. 1:**
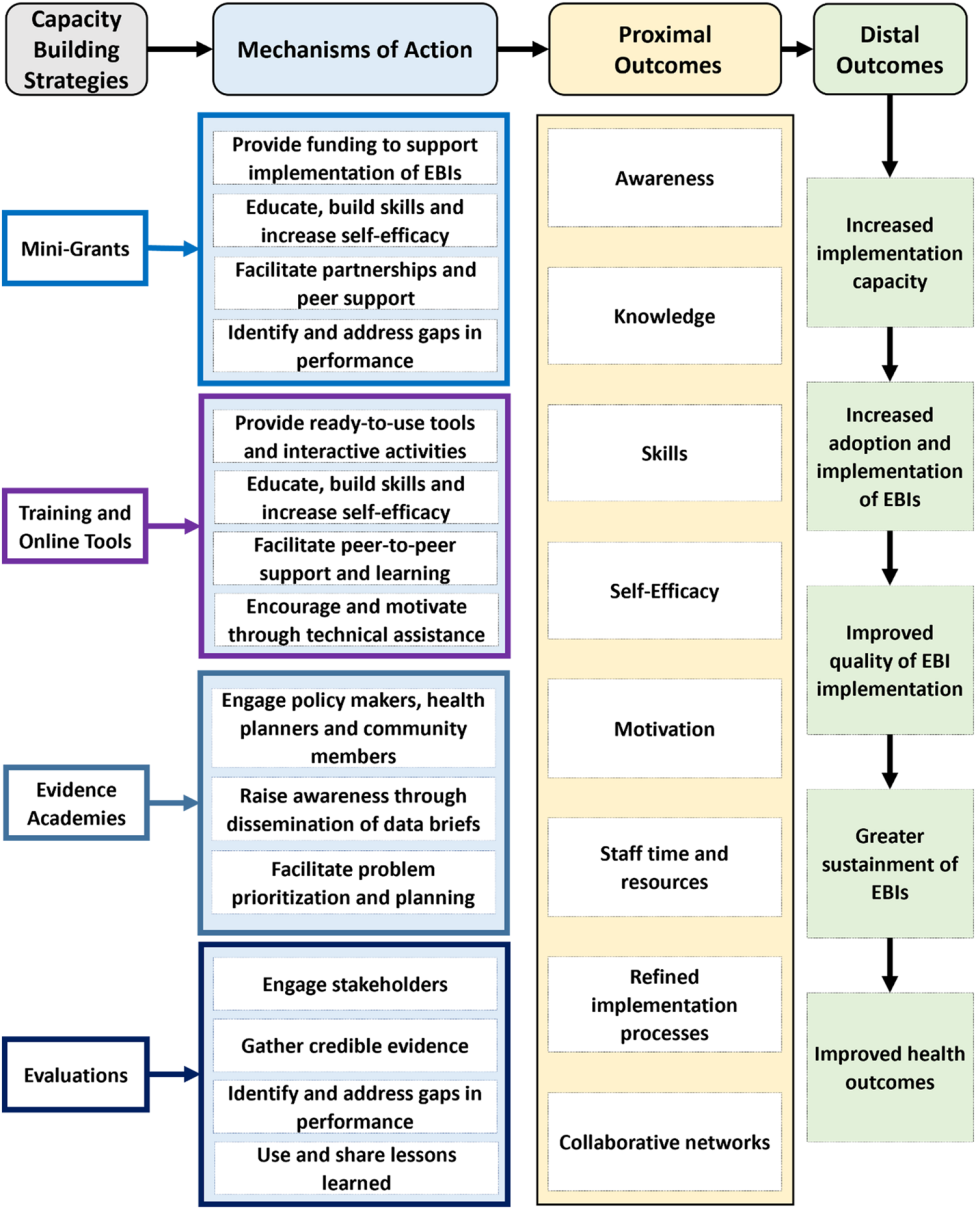
Capacity-building strategies, mechanisms of action, and outcomes

We conducted a narrative review of peer-reviewed publications and grey literature reports of CPCRN projects to develop histories, case studies, and lessons learned for each capacity-building strategy. Figure 1 provides an overview of each strategy, the mechanisms through which they affect capacity, and their proximal and distal outcomes.

## CPCRN capacity-building activities

### Mini-grants

**Fig. 2:**
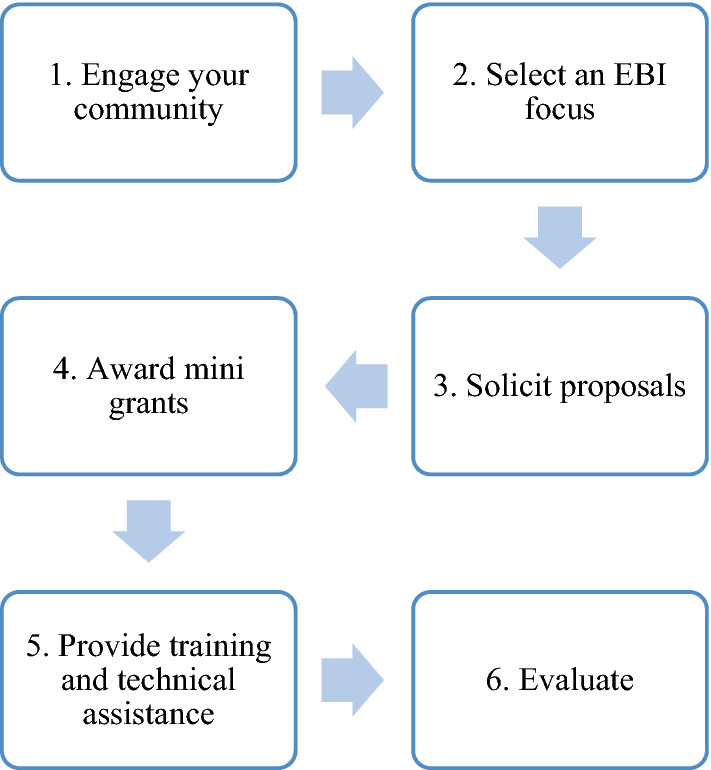
Mini-grant steps

Mini-grant programs provide monetary resources for community partners to incentivize and fund their efforts to implement EBIs [12]. At least six CPCRN centers have used mini grants to promote and support EBI implementation. Mini-grants programs involve the provision of funding (e.g., $10,000) to a community-based organization to support EBI selection and implementation, often coupled with community-engagement, training, technical assistance (TA), and support for evaluation [12]. Figure 2 illustrates the six steps common to mini-grant programs. The Emory CPCRN was among the first centers to use mini grants, using them to increase community-based organizations’ capacity to implement EBIs to address physical activity, healthy eating, tobacco control and other chronic disease prevention efforts since 2007. The first three cycles of the Emory mini grants focused on implementing EBIs on NCI’s EBCCP website [10]. The last round of the Emory mini grants (2012–14) shifted its emphasis to support the implementation of EBIs that focused on organizational policy and environmental change [13, 14]. Learning from Emory, CPCRN centers (Texas A&M, University of South Carolina, University of Texas, University of Washington, and the University of Colorado) have used mini grants ranging from $5,000 to $16,500 to support implementation of EBIs that addressed physical activity, health disparities, and cancer screening [12]. The University of Texas Health Science Center at Houston CPCRN used mini grants to address cancer-related health disparities, particularly among the Lantin(o/a/e). This included a mini-grant to the Cancer and Chronic Disease Consortium of El Paso (CCDC), a small community-based organization that leveraged the experience supported through the mini-grant to then obtain a larger grant from the Cancer Prevention and Research Institute of Texas to adapt and implement an EBI for increasing breast and cervical cancer screening among Latinas [15]. In addition to awarding mini grants, CPCRN centers (The University of Washington and the University of North Carolina) have partnered with mini-grant funders (e.g., Komen Foundation, State Health Department) and have provided training and TA to their grantees.

#### Case study: mini grants—South Carolina

The South Carolina Cancer Prevention and Control Research Network (SC-CPCRN) Mini-Grants Program, referred to as the Community Health Intervention Program (CHIP), began in 2011 and was modeled after Emory’s mini-grants program [12]. The purpose of CHIP is to address cancer-related health disparities and reduce the cancer burden among vulnerable communities in South Carolina. In line with the first step in Fig. 2, the SC-CPCRN engaged its community and research advisory councils to design CHIP and reach out to community partners [16]. Grant recipients are expected to collaborate with a clinical partner such as Federally Qualified Health Centers (FQHCs) or free medical clinics. Most grants have focused on reducing disparities in African American communities [17]. The SC-CPCRN has implemented three rounds of CHIP and provided eight mini-grants to seven community organizations in seven counties (one organization received funding twice). Grantees were directed to the EBCCP website to select EBIs [10]. EBIs have focused on promoting healthful eating, physical activity, breast, cervical, prostate, and colorectal cancer screening, and health screenings more generally, including provision of COVID-19 testing and vaccines. CHIP provided training and TA to grantees as needed. Survey and other evaluation data collected from these community-led initiatives indicate that communities have experienced healthier diets, increased physical activity, improved cancer screening, and increased intentions to be screened for cancer following program implementation [12, 17–19]. Despite the potential for COVID-19 to be a barrier to implementation, most recent grantees (a rural health network and a faith-based organization) recruited and engaged communities successfully due to outdoor and socially distanced programming. Although mini grants are typically one-year in length, many community groups have sustained improvements through ongoing partnerships with SC-CPCRN and, for some grantees, successful applications for additional funding through the statewide cancer alliance. Former grantees provide valuable support for SC-CPCPRN’s work by supporting new CHIP grant recipients, serving on the center’s community advisory council, and partnering on recruitment and dissemination for other health promotion focused initiatives. CHIP grant recipients have also co-published study results with the university investigators and collaborated on community-based cancer prevention and control education [18, 19].

#### Case study: mini grants—Colorado

The Colorado CPCRN started its mini-grant program in the current CPCRN funding cycle (2019–2024), building on the successful mini-grants programs at other network sites. The goal of the Colorado CPCRN’s local project is to increase the use of evidence-based cancer prevention and early detection approaches for individuals at high-risk for cancer, especially EBIs that increase the use of genetic testing and appropriate clinical management strategies as recommended by the Cancer Moonshot [20]. Colorado engaged a 16-member statewide community advisory board with representatives from rural and urban healthcare clinics, community-based organizations, third party payors, and public health agencies. With input from the advisory board, the call for proposals required inclusion of one or more of the following: new partnerships between community and provider organizations; approaches that build on local strengths and address local barriers; multi-level programs that combine activities at the patient, provider, and/or system-level; integration of family history into other ongoing healthcare initiatives such as telehealth and patient navigation. Two organizations were funded at $10,000 each, and work is ongoing in this initial cycle. Both healthcare organizations are in rural and frontier areas of the state with unique population needs, with funds being used for activities across the evidence translation continuum, indicating that the program is supporting a range of capacity-building in under-resourced settings. One healthcare system early in the process of implementation is using the funds to conduct a records review to gather baseline data and begin collecting family history data using the USPSTF guidelines for hereditary breast and ovarian cancer [21]. The second clinic is using the funds to build on ongoing efforts and fully integrate family history into the electronic health records and support new workflows with patient navigation, with the goal of institutionalizing collection and use of family history in routine care.

### Training

**Fig. 3:**
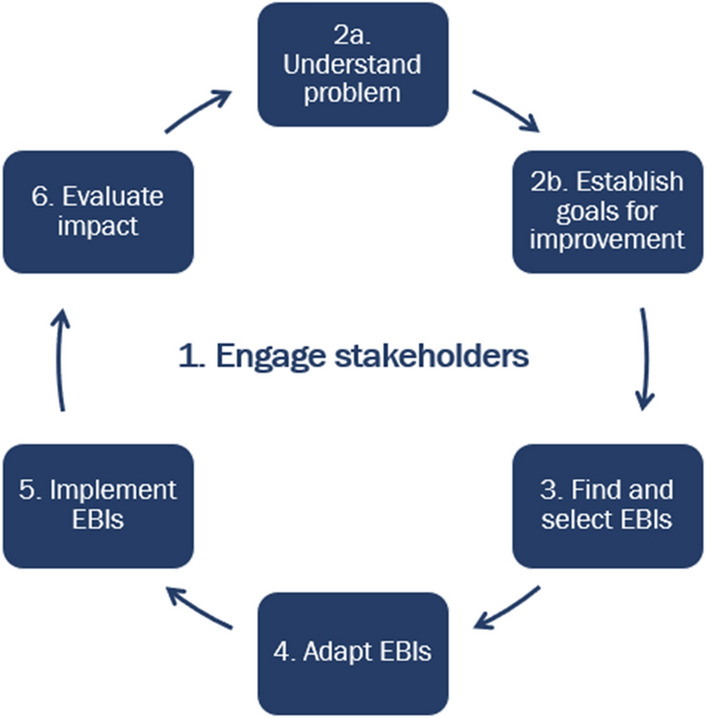
Framework for putting public health evidence in action

During the 2004–2009 CPCRN cycle, investigators from eight centers participated in a Capacity-Building, Technical Assistance and Training (CBTAT) workgroup [1]. The workgroup’s focus was on understanding needs of local practitioners and community partners working in cancer control and building training and tools to help public health and clinical organizations use evidence-based practices. As a supplement to the mini-grants program described above, the CBTAT workgroup developed a training workshop, called *Putting Public Health Evidence in Action* (PPHEIA) that built the skills of funded recipients (community partners) in how to find, select and implement evidence-based programs [23]. At that time, there was limited training on how to implement EBIs in real-world practice and a great need for this knowledge and skill-building [24]. From 2009 to 2014, the CBTAT workgroup focused on updating and tailoring the training for a variety of public health audiences. The members conducted national trainings for the public health workforce by providing workshops on PPHEIA at public health conferences such as American Public Health Association (Learning Institute), Society for Public Health Education (SOPHE), and National Association of County and City Health Officials (NACCHO). They also provided the training to local boards of health and national and regional ACS offices. In subsequent funding cycles, they have revised the training to include additional topics (e.g., quality improvement, and communications). The University of Washington Collaborating Center adapted the curriculum culturally and linguistically for Spanish speakers and has delivered the training to organizations with Spanish dominant employees. The current version includes six modules (Fig. 3). Since 2004, CPCRN has delivered the training, nationally, approximately 25 times to over 1,200 attendees. The training is publicly available on the CPCRN website which contains links to recorded modules, slides, resources, and activities available for download (http://cpcrn.org/training) [22].

#### Training case study: putting public health evidence in action and the CPCRN scholars program

Launched in 2020, the CPCRN Scholars Program was created with the goal of building the implementation science and practice workforce in cancer control. CPCRN invites faculty, graduate students, health professionals and community practitioners to apply to be part of the one-year program during which they participate in trainings and engage in a mentored project. As of September 2022, 37 scholars had participated in the program (Year 1 = 20; Year 2 = 17). Scholars select trainings they will participate in based on their current expertise and future goals. PPHEIA is one of the trainings offered to scholars. To adapt PPHEIA for delivery to a national cohort of scholars, CPCRN members enhanced it to include breakout rooms, polling, chat, and digital whiteboards (e.g., Google Jamboard) [25]. In the first year of the Scholars program, CPCRN members delivered PPHEIA live as a six-part webinar series via Zoom to CPCRN Scholars and other interested CPCRN partners. They recorded the six sessions and uploaded them to the CPCRN website [22] and YouTube channel [26] for future cohorts. Approximately 60 people attended each session. For each session, a range of 13–27 attendees responded to pre- and post-surveys to evaluate their satisfaction with the training and improvements in competencies related to each of the 20 learning objectives across the six sessions. For each of the 20 learning objectives, respondents reported that they had higher levels of confidence in their ability to perform the objectives after the training. Respondents to the PPHEIA evaluation survey requested improvements: additional examples, live demonstrations, and case studies from various settings and more time for discussions, time to practice new concepts, and time to spend in breakout sessions. The Scholars Planning Workgroup has already incorporated areas of improvements in the Scholars Program for future cohorts.

### Online tools for advancing the use of cancer control EBIs

Based on experience collaborating with and training community and healthcare partners, CPCRN centers determined that finding and adapting EBIs remained a challenge for network partners even though some had training in these areas. Through funding from a National Cancer Institute R01 led by UTHealth and Emory University CPCRNs and including an Advisory Committee made up of investigators from several other CPCRN sites, the team developed IM ADAPT [27–29]. IM ADPAT is an online tool designed to help planners find evidenced-based interventions for cancer control and plan adaptations needed to fit within new populations and settings. The tool was developed with input from CPCRN community and healthcare partners and walks users through a process of documenting the needs of a new population and setting, finding an EBI with potential fit, analyzing the selected EBI to thoroughly understand it’s content and mechanisms of action, comparing the existing EBI with the needs of the new population and setting, and then making plans for needed adaptations. Subsequently, based on IM ADAPT, the team developed a tool for clearly describing EBIs for planners who may want to either implement EBIs as intended, adapt them, or develop implementation strategies for delivering them (EBI Mapping) [30]. EBI Mapping has also been used to describe the components and logic of existing CRC screening EBIs from NCI’s EBCCP website [10, 31].

### Evidence academies (EA)

The Evidence Academy Conference Model was developed in 2010 to bring together researchers, health professionals, community members, and policy makers to share the latest EBIs and plan for implementation at the local level. Dr. Cathy Melvin, Principal Investigator of the UNC-CPCRN, envisioned EAs as “a co-learning experience for a relatively small, well-defined network of individuals who represent different sectors but share a collective interest in a specific health priority” [32].

**Fig. 4:**
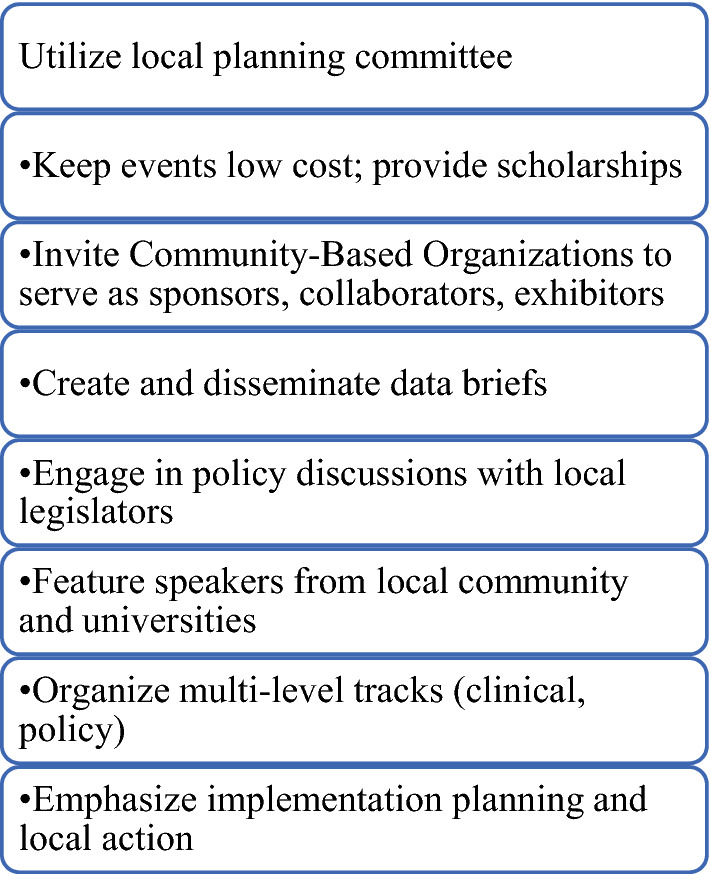
Core components of evidence academies

**Fig. 5:**
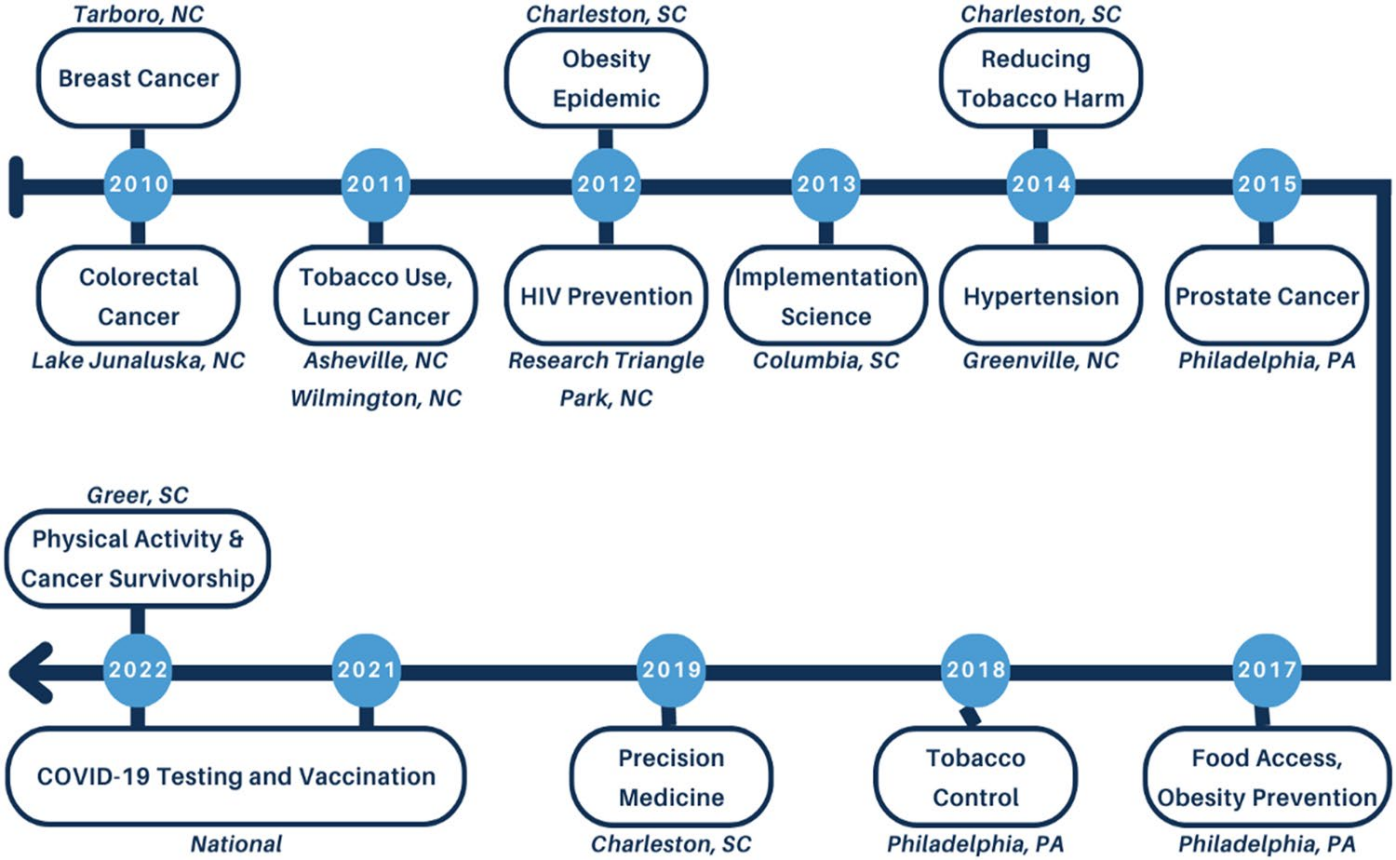
Timeline evidence academy events: 2010–2022

The Evidence Academy topics were initially focused on cancer prevention, screening, and treatment and were held in multiple regions across North Carolina. In 2012, the EA Model was adopted for HIV/AIDS, and subsequent topics included both chronic and infectious diseases. EAs have been held by a CPCRN Collaborating Center in Pennsylvania, taken up by Clinical Translational Science Award grantees in North and South Carolina, and mostly recently sponsored by a large grant from the NIH through the RADx-UP Initiative (Rapid Acceleration of Diagnostics in Underserved Populations). The RADx-UP EA Model focuses on COVID-19, is national in scope, and held on a virtual platform. While the EA model has evolved over time, common elements remain (with a few exceptions for the national RADx-UP Evidence Academies) (Fig. 4). A timeline of EA events is displayed in Fig. 5.

Outside of these common elements, EA planners have made adaptations to the model to fit specific topics, community partners, audiences, and funding opportunities. Early EAs in North Carolina were held in rotating locations across the state and offered CMEs/CEUs to encourage professional organizations to send their employees [32]. The Hypertension EA in North Carolina was the first to be funded by an external grant, which provided resources for implementation activities through a year-long Action Learning Cohort [33]. In Pennsylvania, the Food Access, Diet, and Obesity Prevention EA held a “Public Health Pitch” competition among students to present their ideas on accelerating research and policy [34]. The nationwide RADx-UP EAs prioritize speakers from diverse racial/ethnic communities and all their materials are available in Spanish [35].

A common theme across evaluation results is that the EAs facilitated new partnerships and initiated collaborative projects based on the EBIs presented. The Action Learning Cohort created during the Hypertension EA produced an Empathy-Building Resource Guide for healthcare providers [33]. A group of attendees at a Prostate Cancer EA subsequently collaborated to obtain funding on prostate cancer disparities and community-engagement [34]. The CPCRN has leveraged the EA Model to increase local capacity for cancer prevention and control EBI implementation. Additionally, the model has served as a stimulus for other EA sponsors to create an inclusive space for academic and community members to learn from each other and participate equitably in the research translation process.

#### Evidence academies (EA) case study: reducing the burden of breast cancer

In 2010, the Comprehensive Cancer Control Collaborative of North Carolina (4CNC) focused its inaugural EA in a rural five-county area in the northeastern part of the state. Referred to as “Area L”, this region is home to several state-recognized American Indian Tribes and a large (over 50%) African American population [36]. Community partners were very interested in breast cancer as a topic because incidence and mortality rates were higher in Area L as compared to the state average [37]. Also, women from Area L participate in the Carolina Breast Cancer Study, a population-based case–control study launched in 1993 that is designed to identify causes of breast cancer among White and Black women. At the time of the EA, results had been released that highlighted the urgency of early diagnosis and treatment of the basal-like subtype of breast cancer among younger women, especially African American women. However, analysis of local data also demonstrated a significant breast cancer incidence and mortality gap between African American and White women over 40 living in Area L [38]. These results indicated a need to address disparities across the adult lifespan. The Area L planning committee wanted their community to hear about county-specific statistics, new treatments, genetic testing, and other cutting-edge topics so that local advocacy and service organizations could act upon the new information. The event took place in Tarboro, NC with 83 participants and 15 local and state non-profit exhibitors. The keynote speaker was a breast cancer survivor who founded Sisters Network Triangle, a support organization for Black women with breast cancer. The day concluded with a full group discussion to identify themes and set priorities for future work. EA staff disseminated an evaluation form asking about participant satisfaction with various aspects of the event on a scale from 1 (strongly disagree) to 4 (strongly agree). Fifty attendees completed the evaluation, and the mean scores ranged from 3.37 to 3.59. Participants felt that the EA included opportunities for active learning and the information presented would be useful to their work, specifically:

•“Dissemination of evidence-based education and screening activities in the community”,

•“Methods to help patients have positive and trust-building clinic visits”,

•“More aware of programs in rural areas”, and

•“Data regarding the statistics for eastern North Carolina [and] creating an action plan”.

As described in Bridging Research, Practice, and Policy: The “Evidence Academy” Conference Model [32], 4CNC staff continued to engage with community after the event. A regional cancer coalition subsequently convened strategic planning meetings to work on improving referral systems between counties and across the cancer care continuum. Coalition members developed a cross-county patient resource manual. A follow-up conference was held on the topic of patient-provider communication in the context of rural, African American communities.

### Evaluations of other support systems’ capacity-building initiatives

**Fig. 6:**
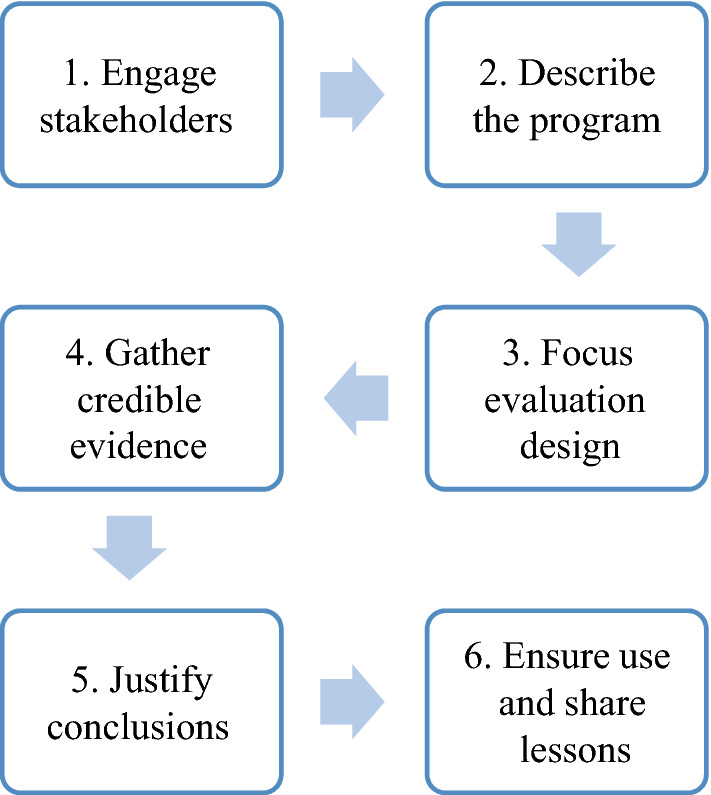
CDC’s framework for program evaluation in public health [39]

CPCRN partners with several other support systems that provide training and other strategies to build practice-level capacity to implement EBIs. These support systems include state health departments, the American Cancer Society, and federal agencies, among others. These evaluations commonly build on the CDC’s framework for program evaluation (Fig. 6) and include the following components: (1) engage stakeholders, (2) describe the program, (3) focus evaluation design, (4) gather credible evidence, (5) justify conclusions, and (6) ensure use and share lessons [39]. In this section we present two case studies of CPCRN evaluations.

#### Evaluation case study: evaluation of the CDC’s colorectal cancer control program (CRCCP)

In 2009, the CDC Colorectal Cancer Control Program (CRCCP) awarded a 5-year cooperative agreement to 25 states and four tribal organizations to increase population-level CRC screening rates to 80% in participating states and tribes and, consequently, to reduce CRC incidence and mortality [40]. CPCRN partnered with CDC to evaluate awardees’ use of EBIs recommended in *The Guide to Community Preventive Services* [41]. A CPCRN workgroup, which included CDC’s Program Evaluation team, led development and implementation of an annual CRCCP awardee survey to assess EBI implementation; this survey was one of several components of the CRCCP evaluation. Awardees increased their use of EBIs over time, and generally used more client-oriented than provider-oriented EBIs; awardees used more EBIs than their counterparts in states without CRCCP funding [5, 42, 43]. CRCCP evaluation findings led to significant changes in the program structure for the 2015–2020 and 2020–2025 funding cycles [44]. CDC now requires all CRCCP awardees to partner with healthcare systems to implement EBIs; many of these partners are FQHCs serving populations experiencing health disparities and low screening rates. The CPCRN workgroup continues to partner with CDC to evaluate various aspects of CRCCP, such as implementation quality of mailed CRC fecal testing programs [45] and the uptake of EBIs and their association with change in CRC screening rates among participating primary care clinics [46, 47]. Over time, the scope of the partnership has grown to include other types of capacity-building activities. For example, they recently developed a toolkit to build awardees’ capacity to assess clinic readiness to implement EBIs and use the readiness data to plan implementation [48].

#### Evaluation case study: evaluation of the American cancer society’s quality improvement learning collaborative for colorectal cancer screening

In 2018, the North Carolina CPCRN (NC-CPCRN) partnered with the American Cancer Society (ACS) and the North Carolina Community Health Center Association (NCCHCA) to evaluate their quality improvement (QI) collaborative. The purpose of the collaborative was to increase nine FQHCs’ capacity to select and implement EBIs for CRC screening through the application of the Institute for Health Care Improvement’s QI methods [49]. Each FQHC identified a three-member implementation team that attended a two-day, face-to-face meeting, or “bootcamp” where ACS staff trained them in QI tools and CRC screening EBIs. ACS-based QI coaches then made monthly calls to support implementation teams as they applied QI tools to select and implement EBIs. 4CNC investigators collaborated with ACS and NCCHCA to design an evaluation plan with a focus on collaborative processes and outcomes, including improvements in CRC screening rates. Results from this evaluation support the positive impact of the collaborative on FQHCs’ implementation capacity and CRC screening rates. All nine FQHCs completed four QI tools, and all implemented CRC screening EBIs (e.g., provider and patient reminders). The FQHCs observed an 8% increase in CRC screening rates in 2018 as compared to 2017. In a post-collaborative focus group, FQHC staff identified barriers and facilitators and illuminated areas for improvement of the collaborative, such as providing more time for peer learning and more support around building capacity to use electronic health record data to drive improvements. Lessons learned were shared back with clinic partners in the form of a data brief and were published in a peer-reviewed manuscript [50]. The partnership built providing evaluation support forged opportunities for future collaborations, including a funded CDC CRCCP for North Carolina [40].

## Conclusions

In these case studies, CPCRN has served primarily as a support system, assisting delivery systems through use of established capacity-building strategies, such as training, technical assistance, tools, peer networking, and funding (e.g., mini grants) [8, 51]. The CPCRN offers numerous lessons learned from their experience with the five strategies outlined in this paper (Table 1). The experiences presented here may be used by future support systems partnering with delivery systems to increase capacity to implement EBIs. Among those lessons emerge three themes, consistent with previous research: (1) community-engagement, (2) partnerships, and (3) co-learning at the levels of the centers, network, and beyond [52].

**Table 1:** Summary of lessons learned across capacity-building activities

Mini Grants	• Engage communities upfront to shape the application focus, disseminate the opportunity, and ensure that the process will be acceptable to and reach the intended groups • Allow flexibility with timelines to allow organizations to apply and implement in accordance with their other planned expenditures (i.e., “rolling applications”) • Keep the scope of the program broad (e.g., allow for multiple types of cancers) to meet the needs of potential applicants • Co-author publications and other products with grantees • Support past grantees and established partnerships to secure funding needed to sustain work beyond the mini-grant’s timeline • Invite previous grantees to serve on the community advisory council, assist with recruitment and dissemination, and offer support to new grantees
Training	• Host live webinars that apply to your audience’s work • Incorporate opportunities for attendees to interact with each other and instructors • Offer support for publication, presentation, grant and other product development • Provide clear expectations of mentorship roles in a training program • Include multiple examples, live demonstrations, and case studies from various settings • Schedule time for discussions, practice, and breakout sessions • Provide culturally sensitive and language concordant resources for organizations with staff members that are not English language dominant
Online Tools	• Define intervention and EBI terms • Design for various levels of user expertise • Include options for practitioners entering at different points in a process (e.g., needing to find an EBI vs already having one to adapt) • Provide variety of helpful tools
Evidence Academies (EA)	• Engage a community advisory committee early in the process • Allow time for planning (9–12 months) • Provide local data on the EA topic in a format that is easy to read and understand • Offer incentives for attendance such as low registration fees, scholarships, dynamic and well-known keynote speakers, and good, local food • Include speaker panels with clinicians and their patients which often result in powerful stories • Seek funding to support implementation of action items collected during the last session of the EA
Evaluations	• Form partnerships early in the evaluation process to guide process evaluations and ensure partners’ preferences are incorporated into evaluation design • Share evaluation results in multiple formats, with all partners • Maintain partnerships beyond evaluation to collaborate on future projects • Use evaluation findings to inform future research studies and projects • Learn from others conducting similar evaluations

Engagement with community partners at the onset and throughout the delivery of a capacity-building strategy is essential for success [53]. In the case of mini-grant programs, upfront community-engagement ensures that the program meets the needs of its partners and that it is acceptable to them. When conducting trainings and hosting Evidence Academies, the intended audiences should be engaged in the preparation so that the content reflects their needs, work, and skill level. Most employees in many organizations serving immigrant communities are dominant in non-English languages. In addition, the UW CPCRN adaptation of PPHEIA curriculum and delivery among Spanish dominant participants demonstrates the increased needs for resources to be culturally sensitive and linguistically concordant to promote health equity.

Support systems conducting evaluations of delivery systems’ programs should engage both the delivery systems and their programs’ recipients in the evaluation design and implementation to ensure that their perspectives are included. The capacity-building strategies used by the CPCRN have resulted in partnerships that have grown and have been sustained beyond the project’s initial timeline. These partnerships have produced co-authored publications (e.g., SC-CPCRN CHIP), secured additional funding (e.g., UNC evaluation of ACS Learning Collaborative leading to funding of NC CRCCP), and developed new programs (e.g., the PPHEIA training comprising part of the CPCRN Scholars curriculum). Implementing capacity-building strategies within a thematic network provides opportunities for co-learning, not just at the center and network levels, but beyond the network, as well.

The knowledge gained from the implementation of the Emory CPCRN’s initial mini-grant program informed the development of additional programs at collaborating centers in Texas, South Carolina and most recently, Colorado. Similarly, the PPHEIA training was developed by the collaborating centers in the CBTAT workgroup, and later adapted and delivered by additional network centers and community partners external to CPCRN. The development of online tools built on the network’s work on training. Last but far from least, the Evidence Academy model started with a focus on breast cancer at a CPCRN center in NC and its use has expanded to additional states, topics, and presently to national-level EAs on prominent issues such as the COVID-19 pandemic. Long-funded thematic networks like the CPCRN can facilitate the transfer of experiences across centers through activities like monthly steering committee meetings that feature local project activities, shared governance, and the gathering of case studies across sites. These activities support the diffusion and improvement of collective capacity-building efforts over time. Future efforts should be directed toward the collective evaluation of the impact these efforts have on community partners’ capacity to select, adapt, implement, and sustain EBIs. These future evaluations might include measures of the proximal and distal outcomes outlined in Fig. 1.

Lessons from the CPCRN’s experience with these five capacity-building strategies demonstrate the value of a thematic research network that has been sustained for twenty years and counting. The communities engaged, the lasting partnerships formed, and the co-learning across and beyond the network were possible in part due to the longevity and the reach of the CPCRN.

## Data Availability

The datasets analyzed for this manuscript are available from the corresponding author on reasonable request.
